# Primary Osseous Leiomyosarcoma of Talus Mimicking Idiopathic Transient Osteoporosis: A Case Report and Literature Review

**DOI:** 10.7759/cureus.84127

**Published:** 2025-05-14

**Authors:** Takashi Katsuo, Takashi Higuchi, Shinji Miwa, Katsuhiro Hayashi, Satoru Demura

**Affiliations:** 1 Orthopaedic Surgery, Kanazawa Red Cross Hospital, Kanazawa, JPN; 2 Orthopaedic Surgery, Graduate School of Medical Sciences, Kanazawa University, Kanazawa, JPN

**Keywords:** idiopathic transient osteoporosis, malignant bone tumor, mri images, primary bone leiomyosarcoma, talus tumour

## Abstract

Primary leiomyosarcoma of the bone (LMSB) is an exceptionally rare malignant bone tumor. We report a case of a 60-year-old woman with LMSB of the talus initially misdiagnosed as idiopathic transient osteoporosis of the talus based on clinical and initial MRI findings, which showed extensive bone marrow edema without soft tissue invasion. Despite initial improvement with off-loading treatment, the patient's symptoms recurred, and subsequent imaging revealed progression and soft tissue involvement. A biopsy confirmed grade 1-2 leiomyosarcoma. Wide resection and reconstruction with a custom-made total talar prosthesis were performed, but local recurrence necessitated a transtibial amputation eight months postoperatively. This case highlights the diagnostic challenge of early-stage LMSB of the talus, which can mimic idiopathic transient osteoporosis on MRI. Clinicians should be aware of this potential pitfall, as the treatment strategies for these two conditions are diametrically opposed: conservative management for idiopathic transient osteoporosis versus prompt surgical resection for LMSB to improve prognosis. Repeated imaging and a high index of suspicion are crucial for timely and accurate diagnosis of this rare and aggressive tumor.

## Introduction

Bone tumors originating in the foot account for 3% of all bone tumors, and those originating in the talus are even rarer, comprising 8-23% of these cases [1]. Leiomyosarcoma is most frequently found in the retroperitoneum, followed by the soft tissue of the extremities and blood vessels, with osseous involvement being a rare manifestation. [2]. As most leiomyosarcomas arise in the uterus or gastrointestinal tract, the presence of such tumors in the bone should raise suspicion of metastasis [3]. Primary leiomyosarcoma of the bone (LMSB) is a very rare sarcoma, accounting for less than 0.7% of all primary malignant bone tumors [4]. Of 136 cases of LMSB observed from 1944 to 2000, only one case (0.74%) involved the talus [5].

In the present report, we describe a 60-year-old woman with a lesion of extensive bone marrow edema in the talus initially diagnosed as idiopathic transient bone marrow osteoporosis of the talus based on clinical and imaging findings. However, progression was observed during off-loading treatment, and an incisional biopsy confirmed that the lesion was LMSB. Thus, the purpose of the present report was to discuss the diagnostic pitfalls and rarity of osseous leiomyosarcoma in the talus through a literature review.

## Case presentation

**Figure 1:**
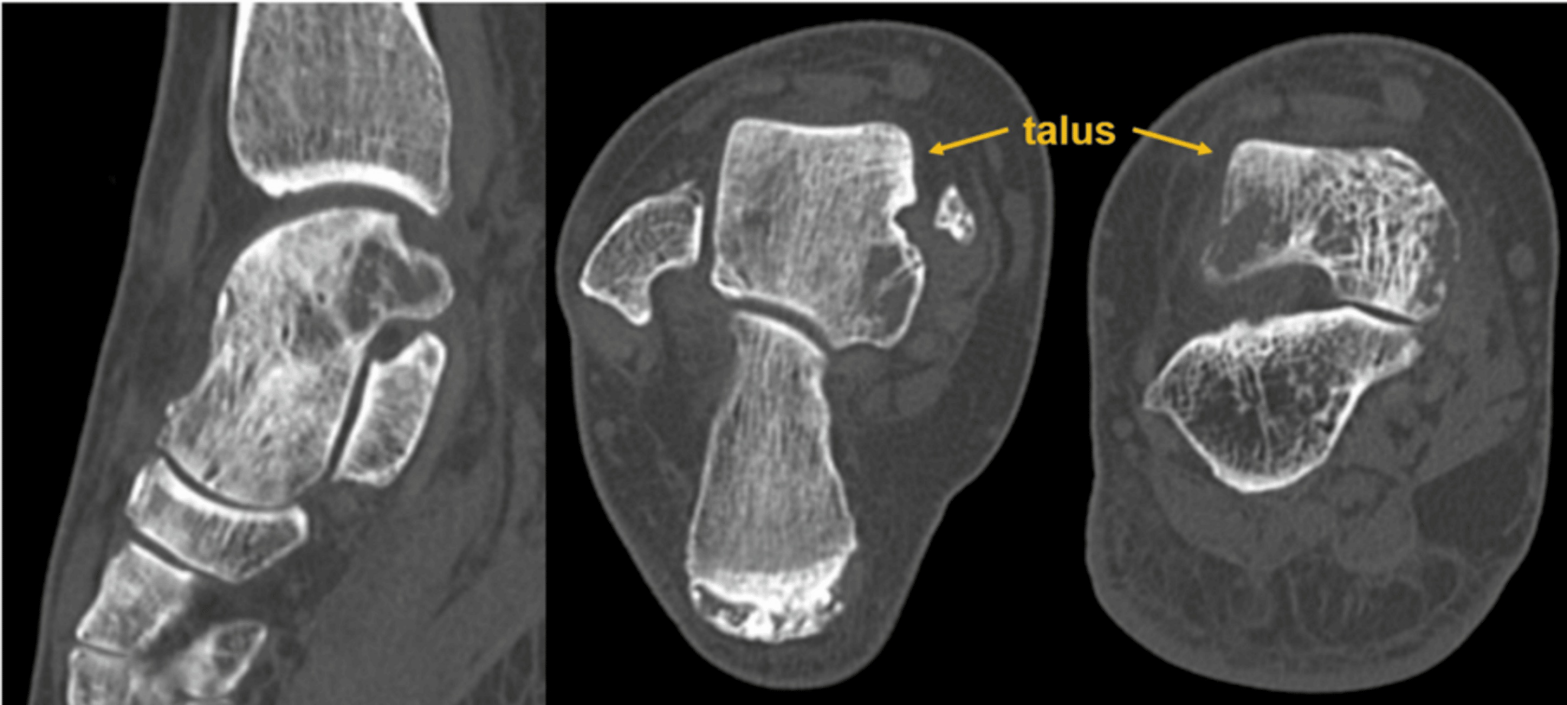
Computed Tomography (CT) scans showed coarse cancellous bone in the entire right talus and some cystic changes.

A 60-year-old female presented to an orthopedic clinic with complaints of right ankle pain which had persisted for a month. Magnetic resonance imaging (MRI) scan revealed signal abnormalities in the talus and she was referred to our hospital for further evaluation. At the initial presentation to our hospital, she reported pain and mild swelling in the right anterior ankle, with minimal pain at rest but tenderness to palpation and exacerbation of pain upon weight-bearing. The patient's height was 163 cm, weight was 88 kg, and Body Mass Index (BMI) was 33. Her medical history included hypertension, and her father had a history of primary macroglobulinemia. X-ray images revealed no abnormal findings including bone injury or osteoarthritic changes. Computed Tomography (CT) scans showed coarse cancellous bone in the entire right talus and some osteolytic changes (Figure 1).

**Figure 2:**
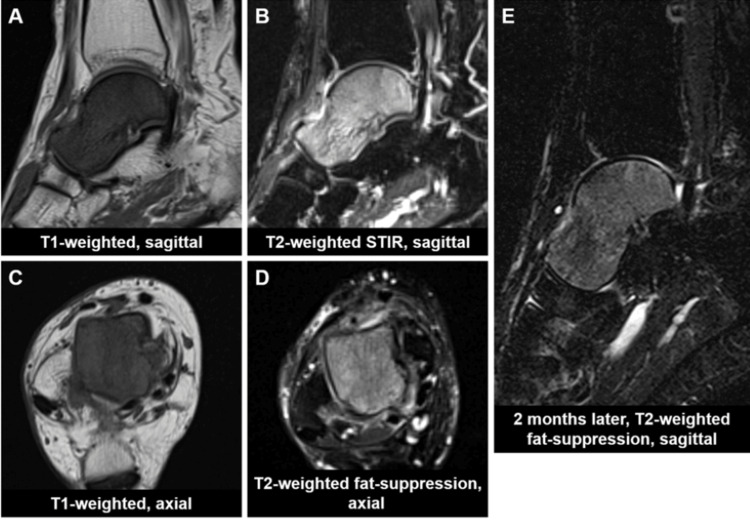
MRI at initial examination and two months after off-loading treatment STIR: short-tau inversion recovery

MRI revealed diffuse hypointensity on T1-weighted images and hyperintensity on T2-weighted images throughout the right talus, with less involvement of the surrounding soft tissue, nor joint effusion (Figure 2A-2D).

**Figure 3:**
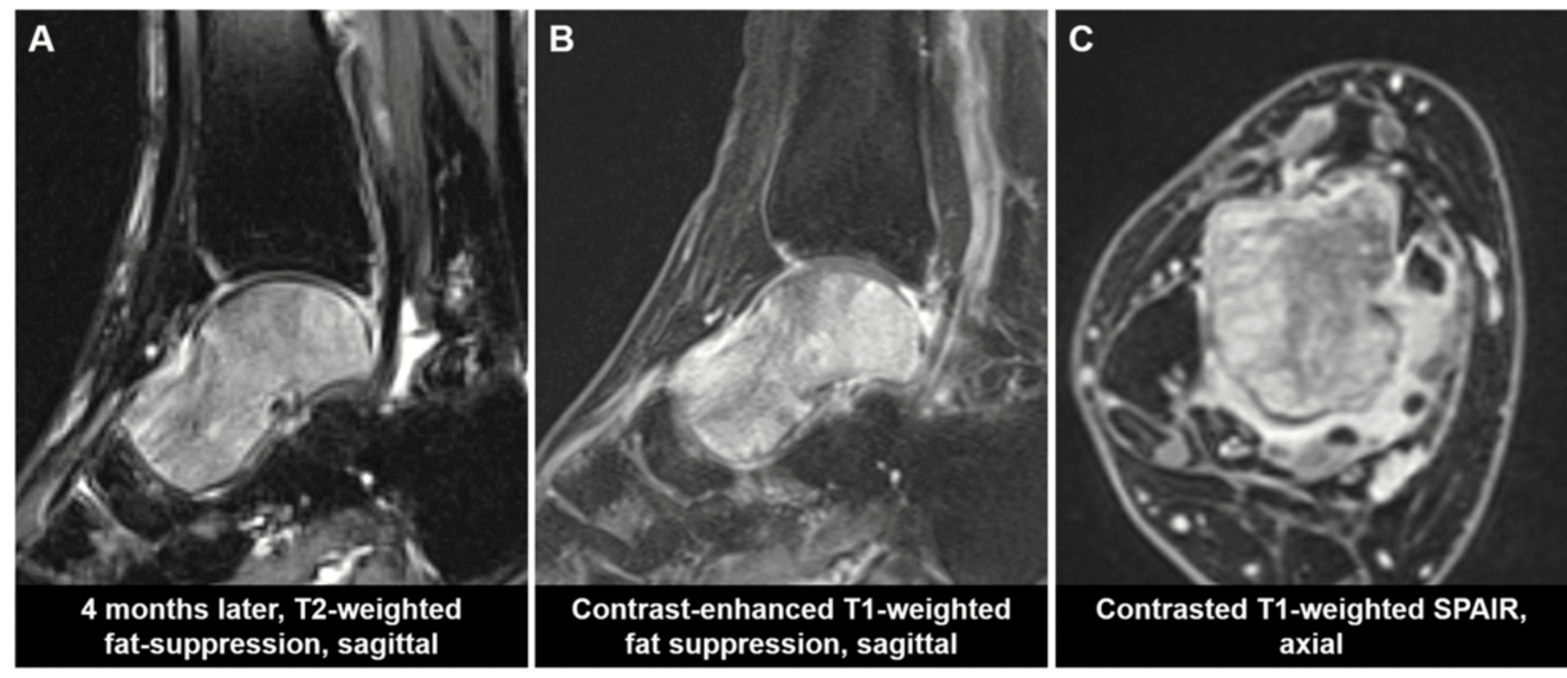
MRI after four months of off-loading treatment SPAIR: spectral adiabatic inversion recovery

The blood test results showed negative tumor markers (alpha-fetoprotein [AFP], carcinoembryonic antigen [CEA], carbohydrate antigen 19-9 [CA19-9], soluble interleukin-2 receptor [sIL-2R]), nor markers associated with multiple myeloma (immunoglobulin [Ig] G, A, M, urinary Bence-Jones protein). The bone metabolism markers (tartrate-resistant acid phosphatase-5b [TRACP-5b], type 1 procollagen N-terminal propeptide [P1NP]) were within normal ranges. Given the MRI findings and the patient's profile as an active, obese woman, a diagnosis of idiopathic transient osteoporosis of talus was considered. The patient was treated with off-loading using a patellar‑tendon‑bearing brace. After two months of treatment, MRI signal changes of the talus, pain, and swelling in the right foot remarkably improved (Figure 2E). However, during another two months of off-loading, pain and swelling in the right foot recurred, and MRI signal changes showed progression (Figure 3A). Consequently, contrast-enhanced MRI was performed, revealing marked enhancement of signal changes in the talus infiltrating the surrounding soft tissue (Figure 3B, 3C).

**Figure 4:**
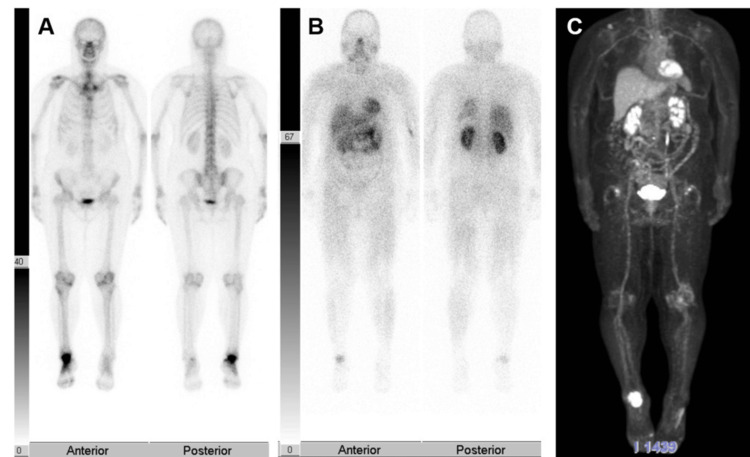
Bone scintigraphy, Thallium scintigraphy, Positron Emission Tomography Computed Tomography (PET-CT)

Upon performing bone scintigraphy and thallium scintigraphy, significant accumulation was observed in the talus, and Positron Emission Tomography (PET)-CT also showed high accumulation with a standardized uptake value max (SUVmax) of 16.3 (Figure 4).

**Figure 5:**
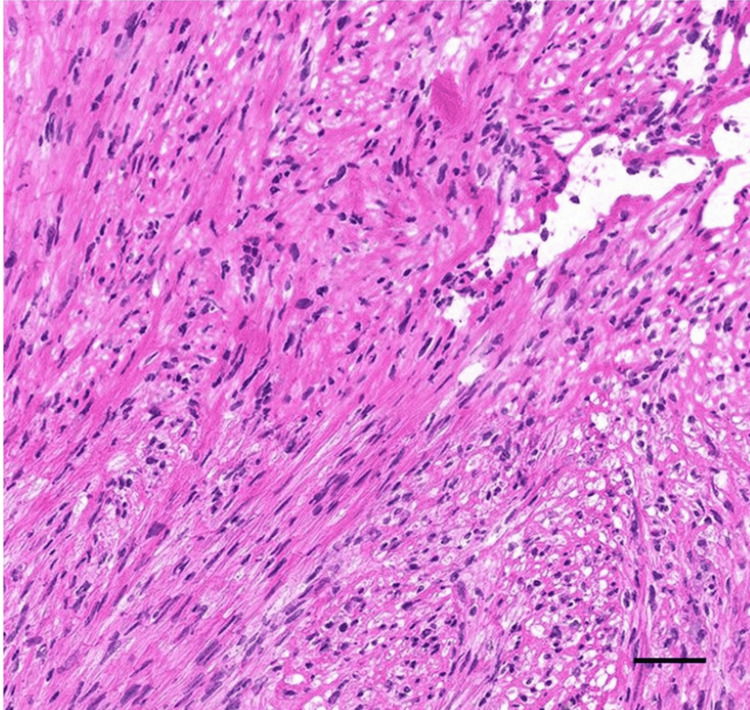
Histology of incisional biopsy

An incisional biopsy was performed, and pathological diagnosis confirmed grade 1-2 leiomyosarcoma with atypical spindle cells and collagen fibers demonstrated positive immunoreactivity for alpha smooth muscle actin (α-SMA), caldesmon, and muscle actin antibody (HHF35) (Figure 5).

**Figure 6:**
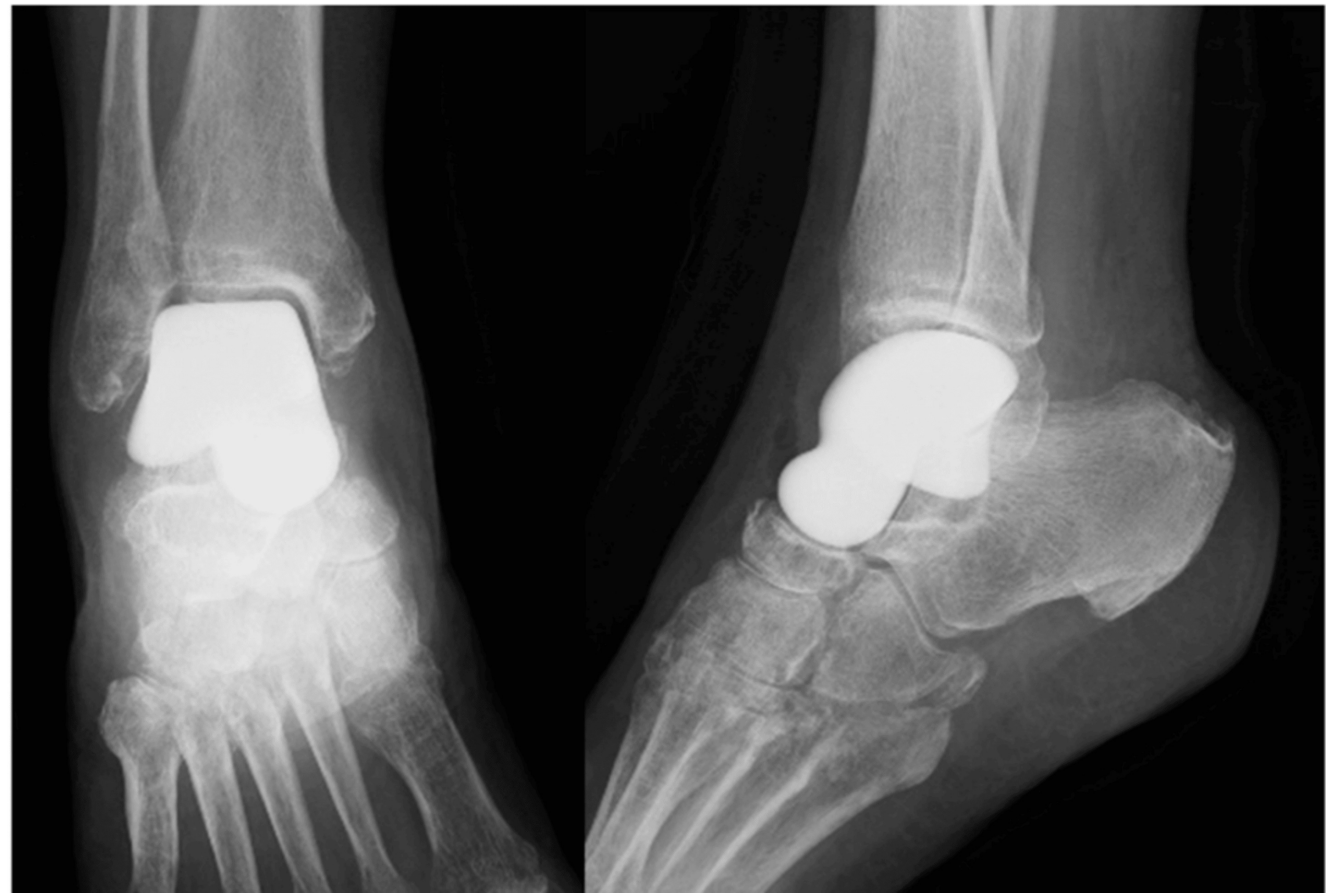
An X-ray image after wide excision with custom-made alumina ceramic total talar prosthesis.

These spindle cells caused extensive cortical bone destruction. Given that PET-CT did not reveal any evidence of primary tumors in other organs, such as the uterus, bone metastasis was ruled out, leading to the diagnosis of primary LMSB of the talus. Following a detailed discussion regarding preoperative chemotherapy, it was not administered due to the patient's wish for a rapid return to her active work. Regarding the surgical approach, the patient, an active laborer, desired limb-sparing surgery despite the possibility of recurrence due to the tumor's proximity to adjacent normal tissues such as muscles and tendons, potentially compromising the achievement of adequate surgical margins. Therefore, wide to marginal resection of the tumorous bone and reconstruction with a custom-made alumina ceramic total talar prosthesis (Kyocera, Kyoto, Japan) were performed (Figure 6).

**Figure 7:**
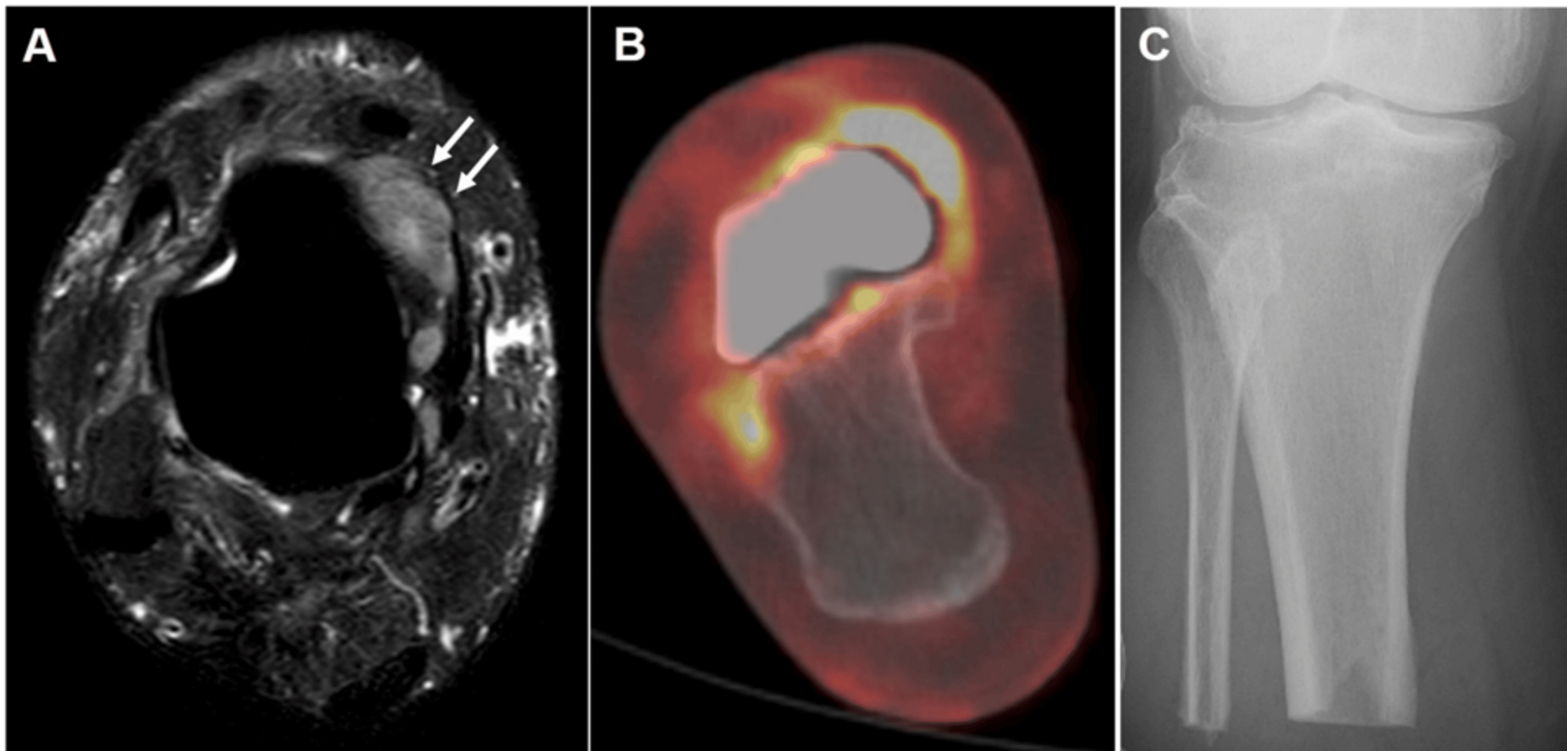
MRI and PET showed the recurrence and a below-knee amputation was performed

As a result of marginal resection, tendon structures were all preserved. Pathological evaluation of the resected tumor revealed that it partially destroyed the articular cartilage, extended to the bone surface, and exhibited dense atypical cells with marked pleomorphism. The final pathological diagnosis was grade 1 leiomyosarcoma of bone, with the resection margin indicating a positive margin. Given the positive resection margin, postoperative chemotherapy and radiotherapy options were discussed with the patient. However, due to her continued strong desire for an early return to her active lifestyle, these adjuvant therapies were ultimately not administered. Follow-up MRI and PET scans revealed local recurrence at the surgical site eight months postoperatively (Figure 7A, 7B). Due to the rapid progression of the local recurrence, a transtibial amputation was performed two months afterward (Figure 7C).

One year and seven months after the initial surgery, and two years and three months after the initial consultation, no further local recurrence or metastasis was observed. The patient was able to ambulate independently with the use of a prosthesis.

## Discussion

LMSB is extremely rare, originating from smooth muscle cells of blood vessels in the bone [5]. It occurs more frequently in women (60%), predominantly in the lower extremity (77%), although involvement of the foot is considerably rare [5-8]. The clinical behavior of LMSB is generally aggressive. The five-year survival rate is reported to be 62-78% and the first-line treatment is surgical resection, due to its poor response to radiation and chemotherapy [7]. Performing definitive surgery with negative surgical margins, following an accurate and early diagnosis before any metastasis, is crucial for ensuring a favorable prognosis [5]. The present report demonstrates an extremely rare case of LMSB of the talus. To the best of our knowledge, the present case represents the third reported case of primary osseous leiomyosarcoma originating in the talus [5,9]. The MRI of LMSB of the talus resembled idiopathic transient osteoporosis. LMSB typically shows low signal intensity on T1-weighted MRI and low to high signal intensity on T2-weighted MRI, and these MRI signal changes can mimic idiopathic transient osteoporosis [10-12]. One notable difference in the present case was the diffuse and intense signal alteration throughout the talus on both T1- and T2-weighted MRI compared to those typically seen in idiopathic transient osteoporosis, however, diagnosis based solely on non-contrast MRI can be challenging. Extensive extraosseous extension of a tumor should strongly suggest the possibility of LMSB or other malignant bone tumors. Among 12 cases of primary osseous leiomyosarcomas, soft tissue invasion was observed in eight cases [10]. In the present case, no soft tissue invasion was observed initially, therefore the MRI findings were similar to idiopathic transient osteoporosis.

Idiopathic transient osteoporosis is a rare clinical syndrome predominantly affecting middle-aged populations, with unknown etiology characterized by the acute onset of pain progressively worsening over several weeks to months [11,12]. The most frequently affected joint is the hip, followed by the knee, foot and ankle, which occur with equal frequency [11]. Transient osteoporosis involving isolated talus, first described by Judd et al. in 2000, is rare; more than 10 cases have been documented in the literature so far [11-13].

The treatment strategies for these two conditions are diametrically opposed [10-12]. While the treatment for idiopathic transient osteoporosis involves conservative treatment with weight-bearing restriction, LMSB requires prompt surgical resection to prevent tumor progression and to improve the prognosis [10-12].

Due to the absence of a cancer history, lack of extraosseous invasion, negative tumor markers, and the extreme rarity of primary bone tumors in the talus, while a bone tumor was considered in the differential diagnosis, it was dismissed in favor of a benign condition. The rarity of primary LMSB of the talus, coupled with the similar MRI findings of idiopathic transient osteoporosis, contributed to the delayed detection of this disease. As with other bone tumors, contrast-enhanced MRI, nuclear medicine imaging, and early biopsy should be conducted for diagnosing malignant bone tumors. Even when idiopathic transient osteoporosis is initially suspected, and symptoms and imaging studies improve with conservative management, repeated imaging studies are important to exclude malignancy. If there is the slightest suspicion of a bone tumor, histologic examination by biopsy is crucial for accurate differentiation and exclusion of malignancy.

## Conclusions

The present case demonstrated an extremely rare primary LMSB of the talus, highlighting the diagnostic difficulty encountered in its early, non-extraosseous extension phase. Its MRI findings resembled those of idiopathic transient osteoporosis, and careful differentiation is required when evaluating talar lesions.
